# Impact of the COVID-19 pandemic on the mental and physical health and overall wellbeing of university students in Portugal

**DOI:** 10.1371/journal.pone.0285317

**Published:** 2023-05-04

**Authors:** Barbara Cesar Machado, Elisabete Pinto, Margarida Silva, Elisa Veiga, Cristina Sá, Sahra Kuhz, Patrícia Oliveira Silva, Ana Pimenta, Ana Gomes, Armando Almeida, Luis Sá, Marta Correia

**Affiliations:** 1 Faculty of Education and Psychology, Research Centre for Human Development, Universidade Católica Portuguesa, Porto, Portugal; 2 CBQF—Centro de Biotecnologia e Química Fina—Laboratório Associado, Escola Superior de Biotecnologia, Porto, Portugal; 3 EPIUnit—Instituto de Saúde Pública, Universidade do Porto, Porto, Portugal; 4 School of Arts, Research Center for the Science and Technology of the Arts, Universidade Católica Portuguesa, Porto, Portugal; 5 Human Neurobehavioral Laboratory, Faculty of Education and Psychology, Research Centre for Human Development, Universidade Católica Portuguesa, Porto, Portugal; 6 Instituto de Ciências da Saúde, Universidade Católica Portuguesa, Porto, Portugal; Polytechnic Institute of Viana do Castelo, PORTUGAL

## Abstract

Throughout the pandemic of COVID-19 caused by SARS-CoV-2, university students were considered a vulnerable risk group for mental health impairment and wellbeing deterioration. This study aimed at evaluating the pandemic’s impact on the physical and mental health and wellbeing among students of a Portuguese university. This cross-sectional study included 913 participants and ran from June to October 2020. Data collected included sociodemographics, three mental health self-report questionnaires (Depression Anxiety Stress Scale, Eating Disorder Examination Questionnaire and Brief COPE) and lifestyle practices (eating and sleeping patterns, media, and entertainment habits) during the first months of the pandemic, which included a 72-day full national lockdown. Descriptive and correlational statistical analysis were conducted. Students’ food habits changed during the pandemic, namely on the consumption of snacks and fast food and, overall, less balanced meals became more prevalent. Additionally, almost 70% of the students reported Body Mass Index changes, while 59% went through sleep pattern changes–these were more pronounced in women and younger students. Over half (67%) of the inquirees exhibited an increase in their stress, depression, and generalized anxiety symptoms. Also, the study demonstrates that students’ lifestyles trended negatively during the pandemic and highlights how important regular psychological, health monitoring and emotional support is, amongst this somehow overlooked population throughout the pandemic. Universities should provide support to overcome challenges in future stressful situations. This study might have an impact on how universities and higher education systems approach their students in terms of mental and physical health monitoring and promotion in future situations, non-related with COVID. Moreover, it has a large sample of students well characterized in terms of mental and physical health, which might be of interest for future comparison with other worldwide group of students throughout stressful situations, such as tragic events, wars, pandemics.

## State of the art

The COVID-19 pandemic has imposed extreme social distancing which abruptly changed routines and impacted existing psychological, diettary, sleeping and physical activity balances [1, 2] and Portugal was not an exception [3]. Since the beginning of 2020 individuals have been forced by various lockdowns to stay indoors, study and work remotely, rethink relationships and reinvent everyday communication. These adjustments have significantly transformed daily habits, such as meal frequency and composition (increased cravings and excess of comfort foods), in an attempt to cope with anxiety, stress and boredom [4]. Studies show that, on average, adult diets suffered a 25% (539 kcal) calorie increase along with a 30% expansion in environmental impact when compared to analogous pre-2020 diets [5].

Although at lower risk for severe complications from COVID-19, 37% of university students were already experiencing some degree of persistent mental distress before the pandemic started, standing out as a group, particularly, at risk for depression and eating disorders [6, 7]. These numbers must not be underestimated, as mental health problems cost global society at least 4.2% of the GDP (Gross Domestic Product) [3].

The pandemic setting added an additional layer of psychological burden, lack of security and predisposition to health impairment [8]. Students are particularly affected by COVID-19-derived financial constraints, estrangement from their emotional support network and uncertainty about graduation and what a COVID-19 tainted future may hold in store [9].

On the physical dimension the pandemic induced a sharp reduction in overall activity, short and long-distance travel, and access to gym facilities. Sedentary behaviors became the new normal with an inevitable cascade of consequences fostering chronic diseases. On top of the natural volatility of their developmental stage, the pandemic has, in fact, placed university students under an unprecedented burden [10], including a deregulation in physiology, homeostasis mechanisms, emotions and humor, resulting in high rates of anxiety with varying (but concerning) levels of stress and depression [11–13].

Given the extent of impacts already measured amongst these students it is appropriate to further evaluate the attributes of those mental health, physical health and well-being trends, as it paves the way to organize and planninginterventions in future similar times, and prevent unnecessary suffering [14–16]

This study aimed to characterize pandemic-related changes in terms of wellbeing, mental health, and general health in university students. The evaluation provides the starting point for the tailoring of a mental health intervention meant to increase literacy and skills to help this group to better deal with the challenges. Additionally, t has been suggested that sex and age influence the general health and mental health amongst university students, hence why, this study, also intends to clarify possible relationships amongst these variables that could help academia to promote tailored-made programs. This effort is part of a broader interdisciplinary program–ACT-19: ACTing on COVID-19 –developed at the Portuguese Catholic University (UCP–Universidade Católica Portuguesa) with its students’ wellness in mind. In particular we wanted to find out how students were coping and whether diet, sleep, mental health, and physical activity patterns had changed.

## Methodology

This is cross-sectional study and adopted a correlational design.

### Data collection

A Qualtrics-based structured questionnaire, prepared for self-administration, was developed and publicized orally and extensive dissemination of emails among students of all four UCP campuses (located in the cities of Braga, Lisbon, Porto, Viseu). No professors or staff were included. Students who did not domain Portuguese language were also excluded. At the time of questionnaire delivery in-person classes had been replaced by online interactions. Portuguese students experienced social isolation for two years (March 2020-April 2022) with a total of 132 days of full national lockdown and mandatory online classes. Of those, 72 lockdown days had elapsed when data collection (which went from June 15^th^ to October 15^th^ 2021) started.

The online survey consisted of three sections: (1) sociodemographics, (2) lifestyle and behaviors (physical activity, dietary habits, sleep patterns, media and entertainment), (3) mental health (perceived anxiety, depression and stress levels, eating disorder behaviors and attitudes and coping strategies). Students were encouraged to think of how the pandemic had influenced them thus far.

Items included in the questionnaire were choosen upon the premise that they would help to determine whether an increase in students’ global levels of stress and anxiety, and overall lifestylechanges had occorred. The items used in the questionnaire included validated tools, such as *Depression Anxiety Stress Scale–DASS-21* [17], the *Eating Disorder Examination Questionnaire–EDE-Q* [18], *Brief COPE* [19], and Kumari *et al*. questionnaire that assess changes in lifestyle-related behavior during the COVID-19 epidemic [20] (the later has been submited for validation within Portuguese population). The questionnaire also included general health questions referring to putative changes of students dietary habits, sleeping habits and tools used to socialize/communicate.

### Instruments and variables

#### 1. Sociodemographics

The first part of survey collected demographic information such as age, sex, educational level, living arrangements and financial and family health concerns.

#### 2. Lifestyle and behavior

This section contained questions based on a similar survey by Kumari *et al*. which aimed to assess changes in lifestyle-related behavior during the COVID-19 epidemic [20]. Additional queries were added to detail the students’ current situation, patterns and experiences including the experience of adversities (e.g., in relationships or finances) and the extent of physical or outdoors activities.

The media and entertainment questions looked into: (1) the main platforms used to communicate, particularly important for the preservation of relationships during lockdown [21] (2) the increase in overall screen time, which is often related to sedentary behavior [22] (3) an overview of recreational screen time, both of social media and streaming/non-streaming entertainment.

Height, body weight and sleep and diet patterns were also collected. The body mass index (BMI) was calculated by the following formula: BMI (kg/m^2^) = weight (in kg) / height^2^ (in m^2^). The World Health Organization defines overweight as a BMI equal to or greater than 25 kg/m^2^ and obesity as a BMI equal to or greater than 30 kg/m^2^ ("Obesity: preventing and managing the global epidemic. Report of a WHO consultation," 2000 *World Health Organ Tech Rep Ser*, *894*, i-xii, 1–253.)

#### 3. Mental health

The mental health section of the questionnaire was structured to assess symptoms through internationally recognized measures.

*Depression Anxiety Stress Scale–DASS-21 [17]*. DASS-21 is a self-report measure consisting of 21 items on emotional symptoms of depression, anxiety, and stress. Each of these dimensions are rated on a 4-point Likert scale, from 0 (did not apply to me at all) to 3 (applied to me very much, or most of the time). Depression scores are considered normal between 0–9, mild depression between 10–12, moderate depression between 13–20, severe depression between 21–27 and extremely severe depression between 28–42. For the anxiety scores the values are: normal between 0–6, mild between 7–9, moderate between 10–14, severe between 15–19, and extremely severe between 20–42. For the stress scores: normal between 0–10, mild between 11–18, moderate between 19–26, severe between 27–34 and extremely severe between 35–42. The Portuguese version of the instrument [23] has also shown good internal consistency, with Cronbach’s alpha values of 0.94 for depression, 0.87 for anxiety and 0.91 for stress [24]. In this study, Cronbach’s alphas were as follows: depression, *α* = 0.93; anxiety, *α* = 0.87; and stress, *α* = 0.93.

*Eating Disorder Examination Questionnaire–EDE-Q [18].* This self-report measure includes 28 items that reflect the number of days, in the past four weeks, when behaviors, attitudes and feelings about eating occurred (e.g., “How many days in the last 28 days did you feel fat?”). These are rated on a 7-point Likert scale, from 0 (no day) to 6 (every day). The combined items create a Global Score and four subscales: Restraint (e.g., “Have you been deliberately trying to limit the amount of food you eat to influence your shape or weight (whether or not you have succeeded)?”), Eating Concern (e.g., “Has thinking about food, eating or calories made it very difficult to concentrate on things you are interested in (for example, working, following a conversation or reading)?”), Weight Concern (e.g., “Have you had a strong desire to lose weight?”) and Shape Concern (e.g., “Have you had a definite desire to have a totally flat stomach?”). The cut-off scores used are: 2.12 for the Global Score and 1.49, 1.37, 2.63 and 2.12, respectively, for the Restraint, Eating Concern, Weight Concern and Shape Concern subscales. The original scale presents good psychometric properties and the Portuguese version of the instrument [25] has also shown good internal consistency, with Cronbach’s alpha for a global score of 0.97. In this study, Cronbach’s alphas were as follows: Global Score, *α* = 0.94; Restraint, *α* = 0.84; Eating Concern, *α* = 0.78; Weight Concern, *α* = 0.85; and Shape Concern, *α* = 0.91.

*Brief COPE [19].* COPE is a self-report measure that consists of 14 scales of two items each, eight of which measure presumably adaptive coping strategies and the other six focus on presumably maladaptive coping. Items include: 1) active coping, 2) planning, 3) using emotional support, 4) using instrumental support, 5) positive refraining, 6) acceptance, 7) religion, 8) humor, 9) venting, 10) denial, 11) substance use, 12) behavioral disengagement, 13) self-distraction and 14) self-blame. Scales 1 through 8 can be seen as adaptive, whereas scales 9 through 14 are possibly maladaptive [26]. Each of these scales are rated on a 4-point Likert scale, from 0 (I haven’t been doing this at alI) to 3 (I’ve been doing this a lot). Carver *et al* [19] reviewed the psychometric characteristics of the Brief COPE and the internal consistency coefficients of all scales were acceptable, although some alpha coefficients were between 0.50 and 0.60 [26] (Meyer, 2001). The Portuguese version of the instrument [27] has shown acceptable internal consistency, identical to the original version. In this study, Cronbach’s alphas were as follows: active coping, *α* = 0.64; planning, *α* = 0.71; emotional support, *α* = 0.83; instrumental support, *α* = 0.78; positive refraining, *α* = 0.78; acceptance, *α* = 0.74; religion, *α* = 0.86; humor, *α* = 0.83; venting, *α* = 0.78; denial, *α* = 0.54; substance use, *α* = 0.92; behavioral disengagement, *α* = 0.77; self-distraction, *α* = 0.69; and self-blame, *α* = 0.71.

### Data analysis

Qualitative variables were described in terms of absolute and relative frequencies (n and %) and then compared using chi-square or exact Fisher test according to the conditions of application of each. Quantitative variables were expressed as mean (M) and standard deviation (SD) or median and percentiles 25 and 75, as suitable. The majority of analyses were conducted comparing younger (<22 years of age) with older (≥22 years of age) students, as well as comparing men and women. Data were analyzed using SPSS version 28.0.

### Ethics commission and confidentiality

The study started with the free and informed online writen consent of students who participated voluntarily and received no freebies, being almost all adults (≥18 years). Survey participants knew data would be used for research purposes only. Their answers were pseudoanonymous and confidential and they were given the opportunity to end their participation in the study and leave the questionnaire at any time prior to submission. The study was approved by the UCP Ethics Commission for Health (Decision 132/2020).

### Results

Responses to the online questionnaire were from students in UCP’s four campuses, encompassing the North, South and Central regions of Portugal and resulted in 913 valid answers.

#### 1. Sociodemographics

Of all participants, 75% were women, with a median age of 22 years (percentile 25; percentile 75: 19; 25) and participants’ age ranged between 17 and 75 years. More than half (59.7%) attended a bachelor’s degree and 32.0% (n = 290) were studying for a master’s degree.

One-fifth (n = 188) of participants were foreigners or displaced from their homes and 10.8% (n = 105) lived alone. Most students (87.4%) classified their house as very good or excellent.

Regarding household income 54.1% (n = 526) reported living comfortably, as opposed to 6.7% (n = 65) that reported it difficult, or very difficult, to live within the current income. Nevertheless, one third (34.7%) refer to experiencing income losses during the pandemic.

Younger students living alone classified their house as better and had a better perception about their familiar income when compared with older students. This characterization is detailed in Table 1.

**Table 1:** Sociodemographic characterization of participants.

	Total n (%)	Age<22Y n (%)	Age≥22Y n (%)	P
**Sex** Women Men *Missings*	692 (76.5) 213 (23.5) 8	351 (80.9) 83 (19.1)	341 (72.4) 130 (27.6)	**0.003**
**Degree** Bachelor Master Other *Missings*	540 (59.7) 290 (32.0) 75 (8.2) 8	400 (92.2) 33 (7.6) 1 (0.2)	140 (29.7) 257 (54.6) 74 (15.7)	**<0.001**
**Displaced students, including foreigners** *Missings*	175 (19.3) 8	67 (15.4)	108 (22.9)	**0.004**
**Students living alone** *Missings*	98 (10.8) 8	32 (7.4)	66 (14.0)	**0.001**
**Classification of your house** Excellent/ very good *Missings*	791 (87.4) 8	402 (92.6)	389 (82.6)	**<0.001**
**Perception about familiar income** Current income allows me to live well Current income is enough to live on It’s hard to live on my current income It’s very hard to live on my current income. Does not know how to judge / prefers not to say *Missings*	465 (54.1) 298 (34.7) 45 (5.2) 13 (1.5) 38 (4.4) 54	259 (63.0) 123 (29.9) 6 (1.5) 2 (0.5) 21 (5.1)	206 (46.0) 175 (39.1) 39 (8.7) 11 (2.5) 17 (3.8)	**<0.001**
**Loss of income during pandemic** *Missings*	300 (34.9) 53	132 (32.1)	168 (37.4)	0.103

#### 2. Lifestyle and behavior

Of all students, 59.5% perceived their health as excellent or very good while 31.0% perceived it as good. These scores were even better among younger participants (66.7% of younger participants vs. 53.1% older students in the good to excellent categories, p<0.001). No statistical differences were observed regarding sex.

When asked to compare their current health to the pre-pandemic period’s, 15.7% reported better health, 51.4% similar health and 32.8% worse health. No statistical differences were observed according to sex or age. Regarding students’ body weight the pre-pandemic BMI was between 13.67 and 45.91 kg/m^2^ (M = 22.99; SD = 4.21) while the pandemic BMI ran between 13.67 and 40.12 kg/m^2^ (M = 22.94; SD = 3.84).

About two thirds (69.0%) of all students changed their body weight since the pandemic started: 36.4% (n = 332) increased their weight, 32.6% (n = 298) decreased it. The remaining answers (16.9%; n = 154) reported weight stability while 14.1% (n = 129) chose not to answer at all.

About one-fifth of all students have experienced COVID symptoms, this proportion being slightly higher among younger students (24.1% in the <22 year old vs. 18.3% in the group ≥22, p = 0.036).

The proportion of smokers was 19.1%, with no statistical differences to be found between men and women. Among smokers 40.9% increased and 20.1% decreased the number of daily cigarettes during the pandemic.

Roughly half of the participants (42.6%) consumed alcoholic beverages, mostly men (60.0% of men vs. 37.3% of women, p<0.001). About 16% of all students increased alcohol consumption during COVID19, while 40.9% decreased it.

The median in sleep time was 7 hours (P25; P75: 7; 8). When questioned about changes in their sleep pattern, since the pandemic, 15.9% (n = 58) of the students reported an increase in sleep time, as opposed to 43.1% (n = 157) who reported a decrease. The latter was more pronounced within women (47.3% vs. 34.5%; p = 0.030) and students aged <22 years old (51.4% in the group aged <22 vs. 35.3% in the group ≥22, p = 0.005).

Aerobic exercise and physical activities increased in 30.2% of all students and decreased in 20.7%. This was accompanied by an increase in leisure and domestic activities during the pandemic in more than 40% of all students.

**Fig 1:**
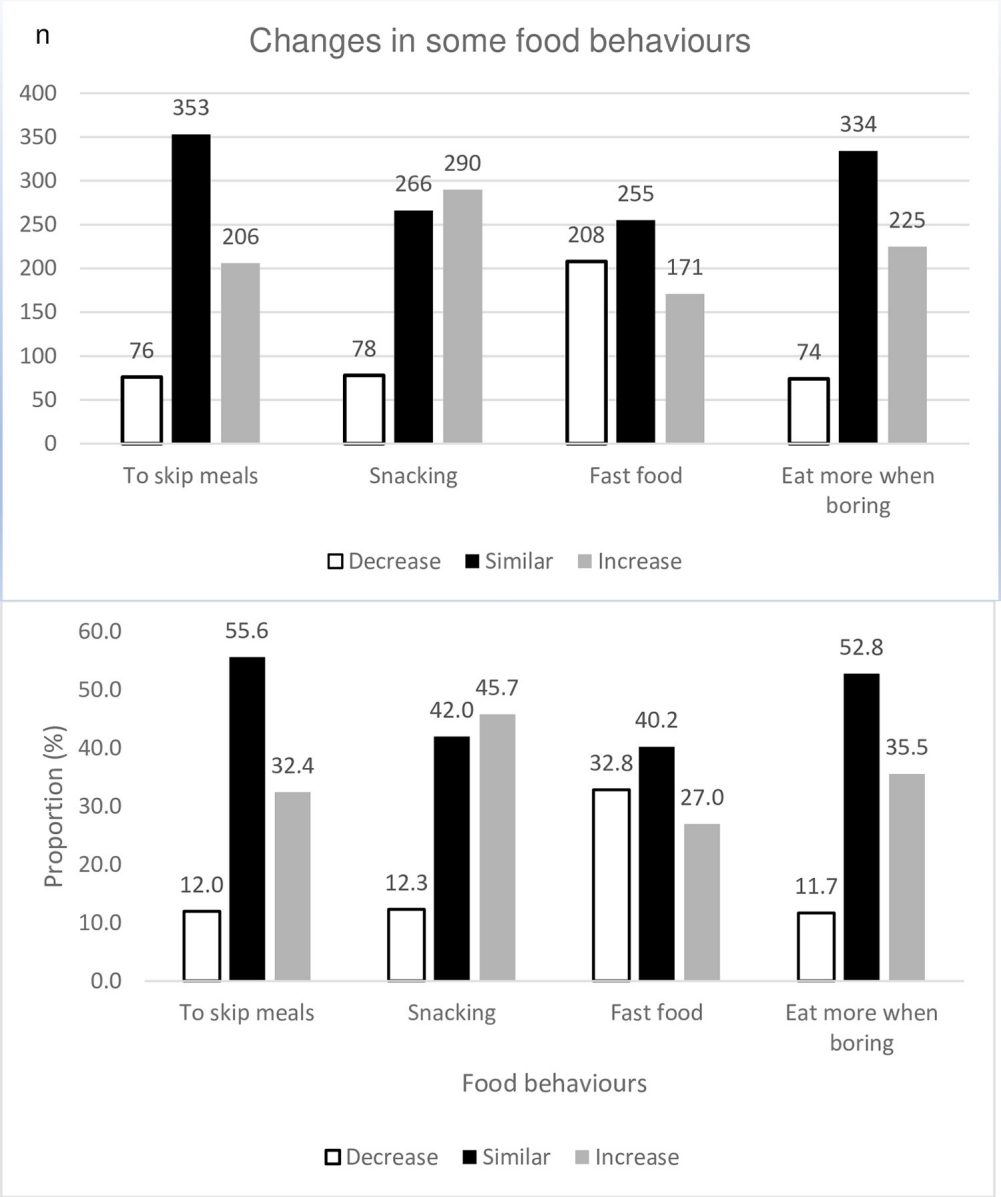


The pandemic also influenced the students’ food behaviors (Fig 1). Students were more prone to skip meals (32.4%), to increase snack consumption between meals (45.7%) and to include more fast-food items, junk food and fries. Additionally, 35.5% reported eating more when bored.

Participants showed ambivalent feelings regarding social relationships during the pandemic: around 21.3% perceived social relationships as excellent/very good, as opposed to 27.5% who considered them bad/not very good. Women perceived relationship quality as having worsened significantly since the pandemic began. The proportion of women that rated their social relationships as “not very good/bad” was 29.2%, compared to 21.5% of men (p = 0.005).

WhatsApp (91%) and mobile phones (77.9%) were the most valued means of communication during the pandemic, with no significant differences amongst students. Most participants increased their daily screen time (93.3%), with no significant differences based on age or sex. This increase in screen time is only partially explained by increased social network usage, which occurred in 79% of participants. Social networks were slightly more appealing to the younger age bracket (85.0% in those aged <22 years vs. 75.1% in the group ≥22, p = 0.001). Of all participants 42% chose videos as their main entertainment source and 29.5% chose music, while other options scored significantly lower (no significant variations observed based on age or sex).

#### 3. Mental health

The proportion of respondents worried about their family’s health because of COVID-19 is 61.2%. Moreover, 66.6% reported an increase in their global levels of stress and anxiety as reported in the “Lifestyle and behavior” section of the survey. Values for DASS-21 scores are presented in Table 2. Close to half the students scored in the normal range for anxiety and depression (49.8%, n = 455 and 48.1%, n = 493, respectively) and the number dropping to 36.9% (n = 337) for Stress. Students were distributed across the remaining severity scores in which the highest values achieved 20% (n = 183) for mild stress, 12.6% (n = 115) for extremely severe anxiety, and 12.2% (n = 111) for moderate depression. Additionally, 9.3% (n = 85) of the students scored in the severe or extremely severe range in the Depression, Anxiety and Stress scales simultaneously. Compared to men, women reported significantly higher mean levels of depression (*t* (779) = -2.04, *p* = 0.042), anxiety (*t* (781) = -2.80, *p* = 0.01), and stress (*t* (782) = -3.72, *p* = < .001) (cf. Table 3).

**Table 2:** Severity of the respondents’ symptoms according to the Depression Anxiety Stress Scale (DASS-21).

	Mean (SD)	n (%)					
DASS-21 scales		Normal	Mild	Moderate	Severe	Extremely Severe	Not Normal (Mild to Extremely Severe)
Depression	10.68 (11.06)	439 (48.1)	96 (10.5)	111 (12.2)	42 (4.6)	93 (10.2)	342 (37.5)
Anxiety	8.56 (9.67)	455 (49.8)	59 (6.5)	109 (11.9)	45 (4.9)	115 (12.6)	328 (35.9)
Stress	15.23 (11.99)	337 (36.9)	183 (20)	114 (12.5)	82 (9)	68 (7.4)	447 (49.0)
Any of the above	34.54 (30.24)	1285 (53.5)	338 (14.07)	334 (13.91)	169 (7.04)	276 (11.49?)	1117 (46.50)

Totals do not always add up to 913 due to missing data.

**Table 3:** Differences between men and women according to the Depression Anxiety Stress Scale (DASS-21).

DASS-21 scales	Sex		*t*
	Men (n = 214) *Mean (SD)*	Women (n = 699) *Mean (SD)*	
**Depression**	9.23 (9.52)	11.13 (11.46)	-2.04 (*p* < .05)
**Anxiety**	6.83 (8.55)	9.10 (9.93)	-2.80 (*p* < .05)
**Stress**	12.38 (10.42)	16.11 (12.31)	-3.72 (*p* < .001)

Table 4 presents the mean EDE-Q global and subscale scores, standard deviations, and the cut-off results as a marker of clinical significance [25]. As can be observed, most of the students scored below the cut-off scores for the Weight Concern, Global Scores, Shape Concern, and Restraint subscales. The Eating Concern subscale was above the cut-off score for 33.2% (n = 303) of the students, the highest rate among EDE-Q subscales. Compared to men, women reported significantly higher mean levels of EDE-Q Global Score (*t* (686) = -3.28, *p* < .001), Eating Concern (*t* (744) = -2.24, *p* = .03), Weight Concern (*t* (689) = -3.53, *p* < .001), and Shape Concern (*t* (745) = -3,70, *p* < .001) (see Table 5).

**Table 4:** Description of the participants according to the Eating Disorder Examination Questionnaire (EDE-Q).

EDE-Q subscales	Mean (SD)	Cutoff scores n (%)	
		≤ cut-off scores	> cut-off scores
**Global Score**	1.24 (1.35)	542 (59.4)	146 (16)
**Restraint**	1.1 (1.39)	467 (51.2)	280 (30.7)
**Eating Concern**	0.69 (1.23)	444 (48.6)	303 (33.2)
**Weight Concern**	1.62 (1.74)	563 (61.7)	184 (20.2)
**Shape Concern**	1.63 (1.71)	517 (56.6)	230 (25.2)

Totals do not always add up to 913 due to missing data.

Note: When the participant’s score falls at or below the cut-off score then his or her functioning is closest to that of nonpatients [25].

**Table 5:** Differences between men and women according to the Eating Disorder Examination Questionnaire (EDE-Q) subscales.

EDE-Q subscales	Sex		*t*
	Men (n = 214) Mean (SD)	Women (n = 699) Mean (SD)	
**Global Score**	.93 (1.05)	1.33 (1.41)	-3.28 (*p* < .01)
**Restraint**	.97 (1.28)	1.13 (1.41)	-1.38 (*p* = .167)
**Eating Concern**	.50 (1.00)	.74 (1.28)	-2.24 (*p* < .05)
**Weight Concern**	1.19 (1.40)	1.74 (1.81)	-3.53 (*p* < .001)
**Shape Concern**	1.21 (1.42)	1.75 (1.77)	-3.70 (*p* < .001)

Finally, Table 6 presents the mean COPE scales and standard deviations organized by their potential adaptive or maladaptive functions. Planning was the adaptive coping strategy that achieved the highest score and, the one that obtained the lowest score, was religion. The maladaptive coping strategy that reached the highest score was self-blame and the one that obtained the lowest score was substance use. Our results also show that, among the 14-coping strategies assessed, the five most used were all adaptative.

**Table 6:** Description of the participants according to the Brief COPE (COPE).

		Mean (SD)
COPE scales	Strategies	
**Adaptive coping strategies**	Planning	2.82 (1.72)
	Acceptance	2.79 (1.68)
	Positive Refraining	2.63 (1.77)
	Active Coping	2.31 (1.56)
	Humor	2.17 (1.74)
	Using Emotional Support	2.09 (1.80)
	Using Instrumental Support	1.86 (1.73)
	Religion	1.37 (1.85)
**Maladaptive coping strategies**	Self-Blame	2.14 (1.74)
	Venting	2.06 (1.69)
	Self-Distraction	2 (1.63)
	Behavioral Disengagement	0.89 (1.31)
	Denial	0.76 (1.15)
	Substance Use	0.28 (0.95)

Totals do not always add up to 913 due to missing data.

## Discussion

Young adults are recognizably vulnerable to mental health problems, as demonstrated by large-scale studies [28]. One of the most striking findings was the high level of stress, depression, and anxiety experienced in more than half of the assessed students. Similar scores were shown in UK students, with a 44% proportion presenting depressed humor and 27% showing anxiety [29]. Other studies have reported prevalence of mental health impairment (anxiety, depression, stress levels) ranging from 17.9% to 89.1%. Radwan *et al*. have shown that Palestinian students presented levels of anxiety and depression above 72.1% [30]; Alyoubi A et al. showed a prevalence of 22% of students suffering from anxiety, 25.4% suffering from depression, and 17.9% experiencing high levels of stress [31]. In Portugal, a study including the general population at the beginning of the pandemic (3-weeks after the first confirmed cases in Portugal), detected the presence of moderate to severe symptoms of depression, anxiety and stress [32] which was shown to be reproductible in university students, too [33].

In addition to the high prevalence of anxiety, stress levels and depressed humor amongst higher education students, which is around 30% [6, 7]., our survey suggests a significant incidence of common mental health problems, in this group. More than half the students signaled levels of stress, anxiety or depression rated between mild to extremely severe. Our results are in line with those published on French university students with a high prevalence of perceived stress, severe depression, and high level of anxiety of 24.7%, 16.1% and 27.5%, respectively [13] The same tendency was observed in Canada where more than half of assessed older adolescents’ and young adults presented an increased stress/anxiety (57.6%) and depression (54.2%) attributed to the pandemic [14]. Likewise, in both studies, female students exhibited higher scores in stress, anxiety and depression, with a greater financial burden, mental health burden, and conflict with parents, when compared to males [34, 35]. Also, a study with Bangladeshi students identified 26.6% and 61.9% of students with mild to extremely severe anxiety symptoms, and depressive symptoms respectively, and 57.1% reported mild to extremely severe levels of stress symptoms, reporting that students’ age and gender, but also family income, residence, and family size were associated with mental health difficulties [6, 35]. Negative perceptions on the effect of the pandemic on life events, mental health, disruptions in education, and health care system, existing physical health conditions, and COVID-19 like symptoms were significantly associated with poor mental outcomes [36].

Our findings are aligned with other recent international studies. A longitudinal prospective study of 217-medical students found that the COVID-19 pandemic affected students mental health with levels of anxiety and stress significantly increased [37]

Another survey shows that higher level of depressive symptoms in women and younger adults are consistent with ours, and also shown higher levels of anxiety symptoms [38]. Indeed, it has been reported that since the pandemic started, mental health issues have been mounting in this age group [39]. It has also been shown that social distancing and other COVID-19 related realities increase symptom severity and psychosocial stress in individuals with self-identified mental disorders [40]. It is likely, therefore, that these changes are linked to the increased levels of anxiety and depression. Interestingly, younger female and more sociable people were described as more prone to experience severe anxiety at the start of the lockdown (quickly decreasing over time) as opposed to younger people with lower incomes and previous mental health problems that have experienced increasing symptoms over time. Although it is well established that the pandemic increased mental health impairment, there are differences in the prevalence of mental health diagnosed problems amongst university students, which might be related with certain pandemic preexisting mental impairment, apart from COVID-19 restriction measures, which entails various levels of affliction in their lives [41]

The COVID-19 pandemic is recognized as a very challenging time that has disturbed the daily life of people everywhere around the world. University students are not an exception as our results have shown, also in accordance with other studies. We believe that the results found related to the reported levels of stress, depression, and anxiety may reflect, in a certain way, the difficulties faced by students during this challenging time and for this reason are not unexpected. Even though, future studies should confirm if the levels of stress, depression, and anxiety are analogous in a non-worldwide stressful period amongst university students. The same for the observed differences between female and male students as our results also supported for a higher mental health compromise among women. Once again, we can speculate and discuss about a pattern of enhanced vulnerability for psychological distress in female students when facing global stressful situations.

Eating disorder behaviors and attitudes were also assessed among the students and our results show that during the pandemic students experienced eating behaviors of clinical relevance. Accordingly, the prevalence rates found for eating concern, restraint, shape concern and weight concern, was between 33.2% and 20.2%, above the cut-off scores. Not surprisingly, and except for restraint, we also found higher scores in the remaining EDE-Q subscales for female students. Our results also supported previous evidence [42] that higher levels of body dissatisfaction are more frequent in females, then in males. Our results also support the notion that the pandemic exerted some influence on student’s food choices. A recent study with university students found a significant correlation between dieting, skipping meals and the risk of eating disorders, in both genders [43]. We are currently trying to deepen the understanding between this association in a further ongoing study. Lifestyle and habit changes, such as a worsening of diet and nutritional status, and a reduction in the amount of physical exercise and outdoors activities, during the pandemic has been evaluated, as shown previously, and our results are in line, reflecting important changes amongst students.

In relation to the adopted coping strategies by students during the pandemic period we have seen a greater use of adaptive coping strategies, compared to maladaptive coping strategies. The five most used coping strategies were all adaptative (i.e., planning, acceptance, positive refraining, active coping, and humor). Recently the effect of coping strategies on anxiety levels in the Italian population during COVID-19 was examined [44]. The authors concluded that emotion-focused coping strategies have a protective effect on both worry and anxiety. Further studies that assess the role of adaptive and maladaptive coping strategies and their association between mental health and mental health deterioration during the pandemic are needed.The study also recognizes the oldest students, expectadly to be postgraduates (>22Y), more complainant with the household conditions and socioeconomic situation. This might be related with the fact that older students have left family households and are currently living alone, facing the expected constraints, as opposed to the youngest students, who are still with their parents or living with flatmates. Nevertheless, most students did not highlight financial constraints as being significant in their lives.

Before the pandemic, the youngest UCP-students perceived their health as better than the oldest students, and again, this might be related with the more pronounced presence of family and financial support amongst the younger ones. Interestingly, post-covid health perception got worse in 32.8% of students; 20% of them had COVID19, and 61% of students were extremely or very worried with family sickness/situation,which might have contributed to the significant increase of anxiety levels (prevalence of 66%)reported by students.

Our survey clearly shows that students are struggling on different levels, most probably due to the very isolated lifestyle situation embraced by them at the time the survey was conducted. Additionally, we are now more able to identify students as the primary target for preventive action following this world pandemic, not only to prevent symptom development, but also to inhibit symptom deterioration. Our study, as others, highlights the traumatic impact of the COVID‐19 pandemic on an already mental health at‐risk group [45], reinforcing the importance of an early detection and intervention in large‐scale disasters.

## Strengths and limitations of this study

The primary strength of this study is the characterization of mental health and identifying important associations, for the first time, in a private university, Portuguese Catholic University (UCP). All the included samples came from an online teaching period and all localities involved had the same level of social distancing. Our sample was significantly high in percentage of women, reflecting a tendency of more women in academia than men, which has been reported in other studies (Browning et al., 2021).

We do not envision any bias related to which students participated in the study questionnaire, since it was available to every student, during the same period of time, and under the same conditions. The survey includes a heterogeneous sample with varying participant ages, educational backgrounds, which we believe makes our results generalizable. The response rate was lower than expected (5–10%), but comparable with observed in other published studies. Additionally, it also provides important characterization of the effect of the pandemic on the students.

This study has some limitations that should be noted. First, the use of a cross-sectional research design that prevents causal inferences and restrains explanations about about pandemic-related changes. Second, and related to the previous limitation, we cannot ignore the potential threat of recall bias and the application of only self-reported measures.

## Conclusion

The study main message emphasizes COVID-19 pandemic substantial impact on students’ a) lifestyle, b) mental health and c) overall health. Indeed, university students are, as described and shown in literature, an already at-risk of mental health impairment group, intensifying underdiagnosed situations or worsening any established mental health symptoms. Additionally, our study also shownthat students have worsen their diet/nutrition and health status, as well as sleeping habbits. Results show a more pronounced negative impact especially concerning a worse diet, more fast-food products and skipped meals, and an increase rate in experiencing negative emotions, high stress levels, anxiety and depression levels. Additionally, this study highlights how social engagement and entertainment experience was affected by the pandemic and supports the widespread notion of how digital forms of communication have assumed a crucial, role during this time frame. It also highlights the utmost importance of universities to take the lead on playing a more active role in identifying students at-risk for mental health, endorsing health promoting programs that might mitigate the burden of this pandemic (or other stressful situation);supporting lifestyle changes, such as physical activity, healthier nutrition, disseminate mental health information and raising awareness on these topics, as well as recommending specialized clinical treatment to at-risk students are all examples of important strategies.

Overall, universities have the potential to deliver tools and resources to improve students’ wellbeing and educational needs, likely to have long-lasting benefits in the post-COVID era.
